# Individual Differences and Similarities in the Judgement of Facial Pain: A Mixed Method Study

**DOI:** 10.3390/ejihpe10040083

**Published:** 2020-12-21

**Authors:** Sheila Glenn, Helen Poole, Paula Oulton

**Affiliations:** 1 School of Natural Sciences and Psychology, Liverpool John Moores University, Liverpool L3 3AF, UK; h.m.poole@ljmu.ac.uk; 2 School of Pharmacy, Liverpool John Moores University, Liverpool L3 3AF, UK; p.oulton@ljmu.ac.uk

**Keywords:** pain, judgement by others, individual differences, similarities

## Abstract

Accurate assessment of pain by health-care professionals is essential to ensure optimal management of pain. An under-researched area is whether personality characteristics affect perception of pain in others. The aims were (a) to determine whether individual differences are associated with participants’ ability to assess pain, and (b) to determine facial cues used in the assessment of pain. One hundred and twenty-eight undergraduate students participated. They completed questionnaire assessments of empathy, pain catastrophizing, sensory sensitivity and emotional intelligence. They then viewed and rated four adult facial images (no, medium, and high pain—12 images total) using a 0–10 numerical rating scale, and noted the reasons for their ratings. (a) Empathy was the only characteristic associated with accuracy of pain assessment. (b) Descriptions of eyes and mouth, and eyes alone were most commonly associated with assessment accuracy. This was the case despite variations in the expression of pain in the four faces. Future studies could evaluate the effect on accuracy of pain assessment of (a) training empathic skills for pain assessment, and (b) emphasizing attention to the eyes, and eyes and mouth.

## 1. Introduction

Pain is a common symptom that motivates people to seek medical treatment. It is a subjective experience and difficult to quantify in others, yet accurate assessment of pain by health-care professionals is essential to ensure optimal treatment or management of the pain. However, there is evidence for inaccuracies in the judgement of pain in others. For example, Davoudi, Afsharzadeh, Mohammadalizadeh, and Haghdoost [1] compared patient and nurse assessments of the intensity of pain in coronary artery disease, using a numerical rating scale. The mean scores of nurses were significantly less than those of patients, but accurate 60% of the time, with 12.4% overestimates and 27.6% underestimates. These results confirm previous findings of inaccurate pain judgements by health professionals [2]; e.g., Kappesser and Williams [3] evaluated 13 studies, finding that health-care professionals were more likely to underestimate pain compared to patients’ relatives.

An under-researched area is how individual characteristics of health professionals may affect their perception of pain in others (e.g., [3,4,5]). It has been argued that empathy to pain in others may affect both assessment and treatment [6]. Empathy arguably has a central role in perception of pain in others, as pain is a special psychological state with great evolutionary significance. Hojat and colleagues developed the Jefferson Scale of Physician Empathy [7] to study the role of empathy in health care. They compared the empathy scores of female nurses, pediatricians, and physicians. They found the former two professional groups to have significantly higher empathy scores than physicians; in addition, large individual variability was demonstrated in all three groups [8]. However, evidence for the effect of empathy on pain assessment is limited [9,10,11]. Preis and Kroener-Herwig [12] in a study with students found that females rated the intensity of pain in others (caused by experimental pressure pain) more highly and also had higher empathy scores. In contrast, Ruben and Hall [13] found that males were more accurate in pain ratings than females, although again empathy showed a significant positive relationship with accuracy of rating. They also postulated that a recent experience of personal pain may lead to more accurate perceptions of pain in others.

A further individual characteristic was highlighted by Sullivan et al. [14], who demonstrated that individuals who scored highly on a measure of pain catastrophizing, also tended to perceive more intense pain in others. This suggests that the perception of pain in others may be linked with the observer’s ability to process sensory information. A paradigm called sensory processing has been proposed [15] to refer to a tendency of some individuals to process a variety of information (including pain) more strongly and deeply than others [16]. Arguably this might affect judgements of pain in others.

Emotional intelligence is increasingly used as a key variable in research related to health [17]. Salovey and Mayer [18] have argued that individuals use emotional intelligence to interact with their environment in a way that takes their own emotions and those of others into consideration, and considerable individual differences were demonstrated in this ability. Thus, emotional intelligence may have a part to play in the perception of pain in others.

In summary, studies of the above personal characteristics have demonstrated individual variation; thus, one aim of the present research was to explore further the roles of gender, empathy, catastrophizing, sensitivity to sensory information, and emotional intelligence in the assessment of pain in others. The second aim was to contextualise such assessments by asking participants to provide reasons for their judgements. We used facial expressions of pain, as they have been extensively employed in studies of pain assessment [4,19].

## 2. Materials and Methods

Design Quantitative and qualitative methods explored the relationship between personal characteristics and the accuracy of individuals’ assessments of pain. Participants completed a questionnaire booklet, then rated the pain expressed in still images of faces expressing no, medium, and high pain. They were asked to give reasons for their judgements.

Participants Following ethical approval from the University Research Degrees Committee, 128 undergraduates (97 F, 31 M) were recruited with fully informed consent. The sample consisted of 38 students of nursing (34 F), 66 of pharmacy (50 F), 16 of social work (12 F), and 8 non-health scientists/engineers (1 F). Ongoing pain in self was reported by 28 of these participants.

**Figure 1:**
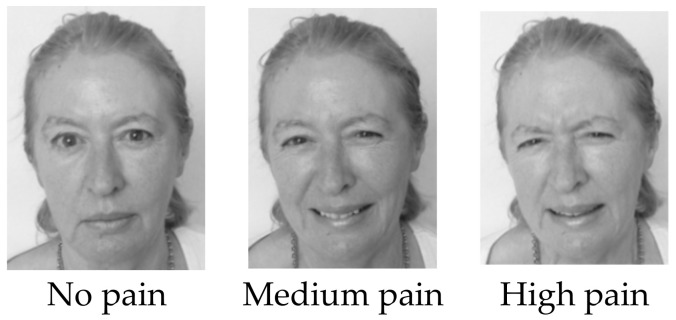
Example of pain faces (Face 2).

Development of Stimuli Images of people experiencing pain of different intensities were developed using the cold pressor task (CPT). The CPT is a well-established experimental technique (e.g., [20]). It involves placing the forearm in iced water (~5 °C) to produce an increasingly painful stimulus that quickly dissipates once the arm is removed. Four volunteers from the research team and laboratory technical team (2 males and 2 females) assisted in development of the stimuli. Upon completion of a health screening questionnaire to ensure they could participate in the CPT, all volunteers gave permission for their images to be used as part of the experimental study. The volunteers verbally rated their pain throughout the procedure on a 0–10 numerical rating scale (NRS) where 0 = no pain and 10 = intolerable pain (i.e., at the point when the pain became intolerable and the forearm was removed from the water). We used these ratings and photographs taken throughout the procedure to develop a range of standardized images of facial expressions of no pain, medium pain, and high pain (Figure 1).

Questionnaire Measures Chronbach’s alphas are given below for the tests in this study. The Jefferson Scale of Physician Empathy [7]. This is a validated self-report 20-item scale rated on a 7-point Likert scale; three factors have been identified: perspective taking, compassionate care, standing in the patients’ shoes. It has been shown to be internally consistent with relatively stable scores over time [8]. Cronbach’s alpha was 0.88. The Pain Catastrophizing Scale [21]. This is a 13-item scale used as a measure of catastrophic thinking in relation to pain; there are 3 dimensions: rumination, magnification, and helplessness. Participants rate their response to pain on a 5-point Likert scale. Good reliability and validity have been reported [21,22,23]. Cronbach’s alpha was 0.92. The Highly Sensitive Person Scale (HSPS) [15]. This is a 27-item scale in which participants are asked to rate their agreement with a variety of statements on a Likert scale ranging from 1 to 7. This scale has been found to have both convergent and discriminate validity [16]. Items reflect sensitivity to a variety of internal and external factors such as noises, life changes, tastes, the arts, fabric, and other people’s moods. Scores can range from 27 to 189, with the higher scores indicating greater sensory processing sensitivity). Cronbach’s alpha was 0.81. The Trait Meta Mood Scale [24]. This 30-item version of emotional intelligence has 3 sub-scales: attention (to feelings), clarity (ability to discriminate between feelings), repair (ability to repair or maintain moods); items are rated on a 5-point Likert Scale. Good internal consistency has been reported. Cronbach’s alpha was 0.82.

Procedure. All questionnaires and procedures were piloted among research team members and a small number of volunteers prior to commencement. The procedure took place in classrooms for the groups of students. Students provided a participant profile: age, gender, experience of current painful conditions in self, and their university course. They then completed the set of questionnaires rating empathy, sensory sensitivity, pain catastrophizing, and emotional intelligence. After a short introduction to the next phase of the study, 12 still images of volunteers demonstrating different intensities of pain expression were shown in random order. The participants were asked to evaluate the faces for pain using a numerical rating scale (0–10) and each participant was then given a sheet with 2 columns; the first was headed “In your own words please describe how much pain the person had”, and the second was headed “What signs did you see that made you think the person was in pain?”. The sessions were delivered to groups of students and lasted approximately 1 h.

Power Analysis. For correlational and multiple regression analysis, Cohen [25] notes that 125 participants provide a power of 80% to detect a correlation at *p* = 0.01, and for a multiple regression with 4 predictor variables, a sample size of 118 provides power of 80% at *p* = 0.1 to detect a medium effect size (0.5). Hence we are confident that the sample size is adequate for the primary analyses.

Statistical Analysis. The Shapiro-Wilk tests for medium and high pain faces were acceptable (*p* = 0.084 and 0.376 respectively). The ratings for no pain faces, not unexpectedly, showed a positive skew, but when 2 outliers were removed, the histogram was near normal. Thus, parametric analyses were used: ANOVA, *t*-tests, Pearson correlations and regression analysis. Bonferroni correction was not employed in order to avoid type II errors in this exploratory study [26].

Qualitative Data Analysis. The text of notes from the students was examined for the strategies and lines of reasoning participants used in making their decisions in evaluating pain. Reasons were assigned to categories, and 2 raters independently checked 13% of the judgements. There was 96.4% agreement.

## 3. Results

Ratings of pain are presented first (Table 1), followed by the personality results (Table 2) and any associations between these and pain (Table 3). Reasons for judgements are then presented. There were no significant differences between the four groups for pain ratings, thus the groups were combined for further analysis. Table 4 and Table 5 show the facial areas that participants were using for their judgements for high and medium pain faces respectively.

### 3.1. Ratings of Pain and Gender/Course Differences

Table 1 shows the ratings given by male and female participants for no, medium and high pain photos.

A two-way mixed measures ANOVA (gender and pain rating) showed a significant effect for pain rating (F_1,124_ = 361.7, *p* = 0.000). Means of 1.6, 4.1 and 5.8 for no, medium, and high indicated the accuracy of these ratings. The main effect of gender was not significant (F_(124)_ = 0.001, *p* = 0.973); there was a significant interaction between gender and rating (F_(1,124)_ = 4.71, *p* = 0.032). It appears that females are more accurate than males on ratings of no pain (i.e., rate lower), and rate similarly to males on medium pain, and slightly higher on high pain.

Self Pain. There were no significant differences between pain ratings (no pain, medium pain, high pain) for those with (*n* = 28) and without experience of ongoing personal pain (F_(1,124)_ = 0.68, *p* = 0.413).

### 3.2. Questionnaire Measures

Table 2 shows male and female scores for empathy, catastrophizing, sensitivity and emotional intelligence.

Gender Differences for Personality Measures. Females were significantly higher than males on empathy (t_(126)_ = 5.79, *p* = 0.000), sensitivity (t_(126)_ = 2.09, *p* = 0.030), emotional intelligence: attention to feelings (t_(126)_ = 2.08, *p* = 0.040), and repair of feelings (t_(126)_ = 2.07, *p* = 0.040). There were no significant gender differences in catastrophizing.

Sensitivity, empathy, and catastrophizing all correlated significantly with one another, but there were no significant correlations with emotional intelligence.

Correlations Between Personality Measures and Pain Ratings. There were only two significant correlations both between empathy and medium pain score (r_(126)_ = 0.19, *p* = 0.032) and high pain score (r_(126)_ = 0.18, *p* = 0.041).

Regression. Females were more accurate at judging whether pain existed or not and empathy was associated with higher pain ratings. We then did a stepwise regression analysis with gender and empathy.

### 3.3. Reasons for Pain Scores

The transcripts were analyzed for the reasons given for judgements. Participants’ comments included:

Medium pain face

*“Eyes almost shut, mouth appears slight grimace not a smile”*

*“Mouth appears slight smile indicating low level pain. Eyes squinting suggesting straining eyes”*

*“Face is drawn in a little like she’s wincing. Eyes shut a little, mouth drawn up”.*

*“The mouth is held funny and the eyes are slits”*

High pain face

*“Eyes nearly shut, looks upset, neck tense, mouth teeth clenched”*

*“Head tilted, eyes squinted, using her mouth to show her pain. Generally looks uncomfortable”*

*“Eyes squinting, frowning, mouth looks as if he is groaning, staring, and concentrating”*

*“Mouth open showing teeth, narrowing of the eyes”*

The above examples show that the eyes and mouth expressions were often mentioned as reasons for people being in pain. There were only a few comments e.g., *“Distress on the face made the pain severe enough to change facial expression”* that were impossible to classify in terms of particular areas of the face.

The comments were then classified as “no reason given”, mentioning “eyes only”, “mouth only”, “both eyes and mouth”, and “whole face with no detail” (the latter were not included in the analyses as they were relatively few and there was no indication of what cues they were using). Table 4 shows the results for the high pain faces 1, 2, 3, and 4. One-way ANOVAS and post hoc Tukey were used to analyze differences between pain scores for these different reasons.

### 3.4. High Pain Faces

Table 4 presents the reasons participants gave for their ratings of high pain faces.

Due to different numbers in groups, four one-way ANOVAS were conducted.

Face 1: There was a significant difference between pain scores (F3,111 = 6.6, *p* = 0.000); those giving no reasons scored pain significantly lower than those highlighting eyes, mouth, and both.
Face 2: There was a significant difference between pain scores (F3,99 = 2.87, *p* = 0.040), but this was due to those who gave no reason having lower scores than those who highlighted specific areas of the face.
Face 3: There was a significant difference between pain scores (F3,112 = 5.62, *p* = 0.001); using both eyes and mouth gave a significantly higher pain score than no reason and mouth only; eyes only was significantly higher than mouth only.
Face 4: There was a significant difference between pain scores (F3,98 = 9.82, *p* = 0.000); again those giving no reasons scored pain significantly lower than those highlighting eyes, mouth, and both.

The greatest number of people gave reasons highlighting both eyes and mouth, suggesting that these are the facial areas on which they concentrate. Numbers are low in the mouth and no reason categories, suggesting caution in the interpretation of statistics. However, those using eyes and mouth on the whole had higher (and therefore more accurate) ratings of pain, followed by eyes alone.

### 3.5. Medium Pain Faces

Table 5 presents the reasons participants gave for their ratings of medium pain faces.

Again four one-way ANOVAS were conducted.

Face 1: There were significant differences in pain rating (F3,113 = 9.89, *p* = 0.000); those using eyes and eyes and mouth gave significantly higher ratings than those using just the mouth or giving no reason.
Face 2: There were significant differences in pain rating (F3,110 = 4.86, *p* = 0.003); those using eyes and eyes and mouth gave significantly higher ratings than those using just the mouth. (Too few n for no reason).
Face 3: There were significant differences in pain rating (F3,86 = 4.22, *p* = 0.008); again those using eyes and eyes and mouth gave significantly higher ratings than those using just the mouth. (Too few n for no reason).
Face 4: There were no significant differences in pain rating (F3,66 = 1.85, *p* = 0.146). Interestingly 58 raters talked about the whole face for this image; e.g., “He looks as though he is trying not to show his pain, concentrating hard to remain expressionless”. Although the different face stimuli produced different rankings, overall eyes, and eyes and mouth are used most often for medium as well as high pain faces. Numbers are low in the mouth and no reason categories suggesting caution in the interpretation of statistics. However, those using eyes and mouth on the whole had more accurate ratings of pain, followed by eyes alone.

## 4. Discussion

The first aim was to investigate the roles of gender, empathy, catastrophizing, sensitivity to sensory information, and emotional intelligence in the assessment of pain in others. Empathy was associated with higher pain scores. This supports the argument of Goubert et al. [6] that empathy affects the assessment of pain in others, and confirms previous studies [12,13]. Females were both more empathic than males in the present study, and also somewhat more accurate in pain ratings; however, it was empathy that was significantly associated with ratings, rather than gender. Previous studies have found inconsistent results for gender (e.g., [12,13]), and it is likely that empathy is the key variable for accurate assessments. Many studies have shown that empathy can be enhanced through interventions and training. For example Batt-Rawden, Chisolm, Anton, and Flickinger [27] reviewed 18 high-quality articles reporting interventions designed to improve empathy in medical students; significant increases in empathy were reported by 15 studies. Cunico, Sartorini, Marognolli, and Meneghini [28] similarly demonstrated that empathy was a skill that could be taught to nursing students.

We found no evidence for a relationship between judgements of pain and other personality characteristics. In particular we did not support a relationship between catastrophizing and ratings of pain in others, even though scores on the Pain Catastrophizing Scale in the present sample are consistent with those of previous studies [21,22]. Such studies [14] have reported a significant relationship, so it is unclear why our results were insignificant.

Of interest, like us, Rubin and Hall [13] found no relationship between personal pain and accuracy of pain rating. However, they found that past experience of a close other’s acute pain was related to accuracy, and this is a measure that should be added in future research.

The second aim was to contextualize such assessments by asking participants to provide reasons for their judgements. Eyes were consistently used as cues for pain judgements. Using both eyes and mouth as facial pain cues was significantly better than neither or one alone for all except Face 3. This was the first high pain face to be presented, and for the later three high pain faces both cues produced significantly higher ratings. Some faces were more expressive than others. Face 4 was the least expressive, but for him both cues produced significantly higher ratings of pain than either cue used alone. Eyes alone were used more than the mouth alone. The implication is that people need to be trained to use both eye and mouth cues in making their judgements and not just rely on either eyes or mouths. The students had at this point received no formal teaching in the judgement of pain, but in spite of this were reasonably accurate as shown by the significant differences in the expected direction between no, medium, and high pain faces. This is important as it suggests that in a clinical situation, with limited time, training on the use of eyes and mouth would be more feasible than the use of detailed coding systems such as the Facial Action Coding System [29] where extensive training and time is required to achieve reliable results.

In the present study (as in previous research), although the high pain images received a significantly higher rating than the other images, the mean was less than the people’s own ratings of pain given at the point the pain became intolerable and they removed their hand from the icy water. A study by McKechnie and Brodie [30] found that although in general health professionals tend to underestimate another’s pain, their sample of lecturers and experienced university educated clinical nurses overestimated patient’s pain. This may relate to the training they had received, and our undergraduate sample had not yet received training.

Limitations. We accept that the images of pain faces used in this study were generated using an experimental (acute) pain task and that the use of static images may have limited individuals’ abilities to rate pain. Available cues from our stimuli were limited when compared to moving images with sound or real-life situations. However, one strength of our stimuli is that pain faces were validated by the volunteers’ own judgements of pain from none to medium to high. There were individual differences in the expression of pain among the stimuli; some faces were more expressive than others, and easier to rate; e.g., “*His features don’t seem to change really; Looks like he’s deep in thought, distracted and concerned. No eye contact, trying to distract himself”.* As can be seen from Table 5, Face 4 had fewer specific comments than the other faces. Similarly, there would be individual variations in the facial expression of pain in real life (rather than laboratory) situations. Facial expression has been shown to be an important factor in real life situations. For example, Igier, Sorum, and Mullet [31] compared facial expressions, verbalizations, avoidance of movement, and interpersonal contact in the assessment of pain in elderly patients. Three conditions were used: where the illness was not known, arthritis, and cancer. For all health conditions, facial expressions were the most important cue for judging pain; this was particularly the case when the illness was not known.

A further limitation is the use of undergraduate students, 94% of whom were studying health and social care courses. They may already have had sensitivities to pain that may have reflected their choice of courses.

Future studies could recruit a sample from the general population to determine whether the use of eye and mouth in pain judgement is universal. Evaluating the effects that training in both pain assessment and increasing empathy have on individuals’ abilities to rate pain accurately would also be informative.

## Figures and Tables

**Table 1:** Gender differences in pain ratings—min. and max. added.

	Participant Gender	Mean	Std. Deviation	N	Min.	Max.
Total no pain	Female	1.52	1.10	96	0	7
	Male	2.03	1.24	30	0	6
	Total	1.64	1.15	126	0	7
Total medium pain	Female	4.14	1.24	96	1	5
	Male	4.04	1.34	30	1	7
	Total	4.13	1.26	126	1	7
Total high pain	Female	5.87	1.56	96	2	10
	Male	5.48	1.81	30	1	8
	Total	5.78	1.62	126	1	10

**Table 2:** Mean empathy, catastrophizing, sensitivity, and emotional intelligence.

Participant Gender		Empathy	Catastrophizing	Sensitivity	Emotional Intelligence
Female	Mean	115.5	34.9	116.9	110.6
	N	97	97	97	97
	Std Dev ^n^	13.2	11.9	16.5	11.7
Male	Mean	99.9	33.3	109.4	106.1
	N	31	31	31	31
	Std Dev ^n^	12.6	8.3	20.1	11.0
Total	Mean	111.6	34.5	115.1	109.5
	N	128	128	128	128
	Std-Dev ^n^	14.7	11.1	17.6	11.6

^n^ number of participants in each group.

**Table 3:** Regression of gender and empathy score on medium and high pain.

Mode	**Standardized Coefficients**	Beta	t	**Sig.**	**95.0% Confidence Interval for B**	
					**Lower Bound**	**Upper Bound**
1	(Constant)		2.326	0.022	0.332	4.140
	Empathy	0.193	2.150	0.034	0.043	1.055
Dependent variable: medium pain.						
Mode	**Standardized Coefficients**	Beta	t	**Sig.**	**95.0% Confidence Interval for B**	
					**Lower Bound**	**Upper Bound**
1	(Constant)		2.824	0.006	0.943	5.368
	Empathy	0.236	2.665	0.009	0.203	1.379
Dependent variable: high pain.						

For both medium and high pain faces, empathy was the only significant associate (R^2^ = 0.027, R^2^ = 0.038 respectively).

**Table 4:** On a scale of 0 to 10 how much pain did you think this person was showing (mean and SD)?

High Pain	No Reason	N % Age	Eyes Only	N % Age	Mouth Only	N % Age	Eyes and Mouth	N % Age
Face 1	3.5	6 5.20%	7.1	28 24.3%	7.3	10 8.60%	7	71 61.70%
Face 2	4.0 (2.4)	6 5.80%	6.1 (1.8)	30 29.10%	6.3 (2.4)	7 6.80%	6.2 (1.6)	60 58.20%
Face 3	3.4 (3.1)	7 6.00%	5.6 (2.3)	24 20.70%	3.7 (2.6)	9 7.80%	6.0 (2.0)	76 65.50%
Face 4	0.8 (0.9)	7 6.90%	4.4 (3.4)	14 13.70%	4.0 (2.2)	25 24.50%	5.7 (2.4)	56 54.90%

**Table 5:** On a scale of 0 to 10, how much pain did you think this person was showing (mean and SD)?

Medium Pain	No Reason	N % Age	Eyes Only	N % Age	Mouth Only	N % Age	Eyes and Mouth	N % Age
Face 1	0.3 (0.7)	9 7.70%	3.5 (1.7)	74	1.0 (1.4)	2	3.5 (2.1)	32
Face 2	3.0 (3.6)	4 4.40%	6.3 (2.2)	22 24.40%	3.7 (3.1)	15 16.70%	5.5 (2.3)	49 54.40%
Face 3	1.5 (2.1)	2	4.3 (1.5)	29 25.40%	2.7 (2.3)	19 16.70%	4.5 (2.1)	64 56.10%
Face 4	2.0 (2.8)	5 7.10%	3.3 (2.1)	44 62.80%	3.9 (2.9)	7 10%	4.6 (2.6)	14 20%
